# Smartphone and Tablet-Based Sensing of Environmental Radioactivity: Mobile Low-Cost Measurements for Monitoring, Citizen Science, and Educational Purposes

**DOI:** 10.3390/s19194264

**Published:** 2019-10-01

**Authors:** Oliver Keller, Mathieu Benoit, Andreas Müller, Sascha Schmeling

**Affiliations:** 1 CERN, Esplanade des Particules 1, 1217 Meyrin, Switzerland; 2 Section de Physique and Institut Universitaire de Formation des Enseignants (IUFE), Université de Genève, 1211 Genève, Switzerland; 3 Département de Physique Nucléaire et Corpusculaire (DPNC), Université de Genève, 1211 Genève, Switzerland

**Keywords:** natural radioactivity, radon, terrestrial radiation, silicon sensor, hybrid pixel detector, formal and informal learning, citizen science, learning tool, open educational resource, low-cost

## Abstract

Sensors for environmental radioactivity based on two novel setups using photodiodes, on the one hand, and an advanced tablet-based hybrid pixel detector, on the other hand, are presented. Measurements of four kinds of terrestrial and every-day radiation sources are carried out: Airborne radon, a mineral containing traces of uranium, edible potassium salt, and an old radium watch. These measurements permit comparisons between different types of ambient radioactive sources and enable environmental monitoring. Available data comprise discrimination between α- and $\beta^{-}$-particles in an energy range of 33 keV to 8 MeV and under ambient air conditions. The diode-based sensor is particularly useful in portable applications since it is small and sturdy with little power consumption. It can be directly connected to a smartphone via the headset socket. For its development, the low-cost silicon positive-intrinsic-negative (PIN) diodes BPX61 and BPW34 have been characterised with capacitance versus voltage (C-V) curves. Physical detection limits for ionising radiation are discussed based on obtained depletion layer width: (50±8) μm at 8 V. The mobile and low-cost character of these sensors, as alternatives to Geiger counters or other advanced equipment, allows for a widespread use by individuals and citizen science groups for environmental and health protection purposes, or in educational settings. Source code and hardware design files are released under open source licenses with this publication.

## 1. Introduction

Measuring radioactivity and ionising radiation is a broad topic relevant in many commercial and research fields, as well as for the living environments of citizens. In non-professional contexts (education, citizen-science), measurements are typically based on Geiger-Müller detectors [1,2,3], which cannot discriminate different types and energy spectra of the analysed radiation. They are limited to measure intensities in the form of count rates, which is just sufficient to roughly compare the activity of radioactive sources and estimate potentially hazardous levels of exposure. In order to enrich the available toolkit and foster better insight on natural radioactivity we propose two variants of solid-state detectors for ionising radiation based on silicon sensors. Appropriate experiments for detecting terrestrial radiation and every-day sources of radioactivity for both detector types are discussed below. The first detector is based on affordable and widely available photodiodes for general use and can be operated together with a smartphone or tablet employing the headset connector. The second one is based on advanced hybrid pixel detector technology [4] and uses means of augmented reality in combination with a mobile tablet. Since pixel detectors are a matrix made of semiconducting diodes, the other detector based on discrete photodiodes serves, as well, as a simplified functional model of one pixel.

With digital cameras integrated in modern smartphones, their sensitivity to radiation beyond the visible spectrum has been exploited to use them as detectors for ionising radiation in physics education settings [5] and radiation dosimetry applications [6]. However, with pixel dimensions in the order of a few micrometers, these sensors embedded in mobile consumer devices lack sensitivity towards ionising radiation and can barely discern different types and energies. The most promising measurements reported suggest discrimination between only two categories: traces of minimum-ionising particles like atmospheric muons and other types of terrestrial radiation (α,β,γ) as a whole [7,8]. The versatility of such a detector is, therefore, comparable to a Geiger-Müller counter and limited to measure relative radiation intensity across long measurement intervals (10 min to 25 min are recommended in [6] even for high ambient dose rates of 10 μSv/h). Moreover, since corresponding smartphone applications must take individual properties of the specific imaging sensor into account, the measurement accuracy is questionable if the software does not provide calibration settings for specific smartphone cameras. Technical limitations, such as hot pixels and varying dark noise thresholds [9] caused by continuous miniaturisation trends, require reproducible and standardised calibration of imaging sensors. For spectrometric measurements in the visible range of light, [10] released recently open source software in order to obtain calibrations und promises to maintain an open database. We are not aware of such efforts in the context of detecting ionising radiation where this is even more important since smartphone cameras are not designed at all for this purpose. Furthermore, regular updates of the application are necessary in order to compensate for changes in the mobile operating systems. For example, the mobile application used in [5] has not been updated for iOS devices in the last three years and does not work properly with current iPhones and iOS versions (last time checked: 3 September 2019). From the six applications investigated in [6], it is the only application which is still available on the app store. Our approach is based on external radiation sensors with well known characteristics which are accompanied and enhanced by mobile open source software. Establishing a high level of compatibility and re-usability also on future mobile platforms is a major concern throughout the presented project.

## 2. Materials and Methods

**Figure 1:**
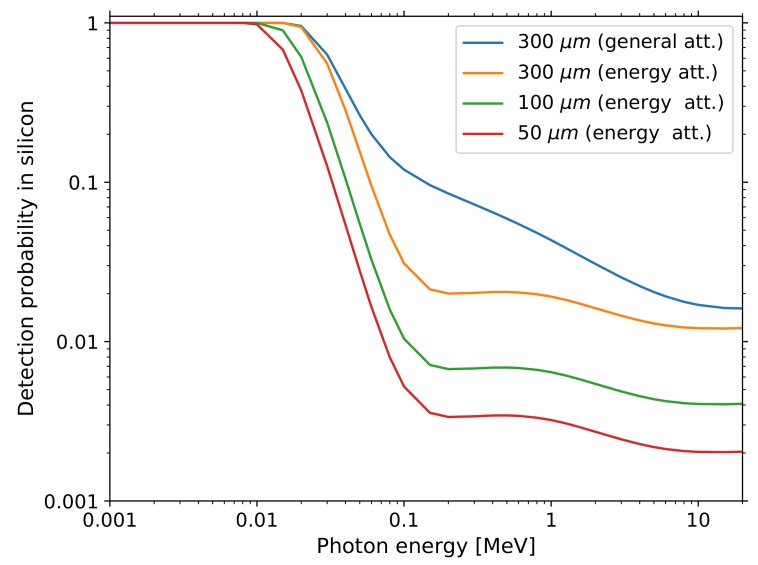
Detection probabilities for photons in 50 μm, 100 μm, and 300 μm thick silicon: The top **blue** line is derived from the general attenuation coefficient μ. The three curves below are based on the energy attenuation coefficient $\mu_{en}$, which results in even lower probabilities. It takes into account that only a fraction of the photon scattering results in ionisation of sensor material and is able to form a detectable electrical signal. The coefficients are extracted from the NIST database [13].

The radiation sensors used in this work are based on silicon semiconductors. Their thin sensitive layer only absorbs certain types and energy ranges of ionising radiation completely and interacts partially or not at all with X-rays or γ-photons. Figure 1 shows the detection probabilities of photons in thin layers of silicon across a wide energy range. For thin layers of 100 μm and less, which is typical for the investigated photodiodes, less than 1% of photons form a detectable signal if their energy is greater than 100 keV. The presented diode sensor characterisation, detector design and suitable experiments are focused accordingly on the measurement of α-particles and electrons from $\beta^{-}$-decays. Taking into account the low activity of investigated sources of radiation, contributions from X-rays and γ-photons can be generally neglected in the detected signals.

Heavily ionising radiation, such as α-particles, interacts strongly and is, therefore, fully absorbed within a few tenth of micrometers [11]. Lighter charged particles, such as electrons, are absorbed up to a maximum energy value per traversed sensor material of about 1 keV/μm to 1.5 keV/μm [11,12]. The absorption of ionising radiation leads to liberated charges in the sensitive volume of the silicon: on average 3.64 eV of ionisation energy create one electron-hole pair. These charges form a small current pulse across the sensor chip that can be amplified and converted into a voltage pulse which is proportional to the deposited energy.

### 2.1. DIY Particle Detector

**Figure 2:**
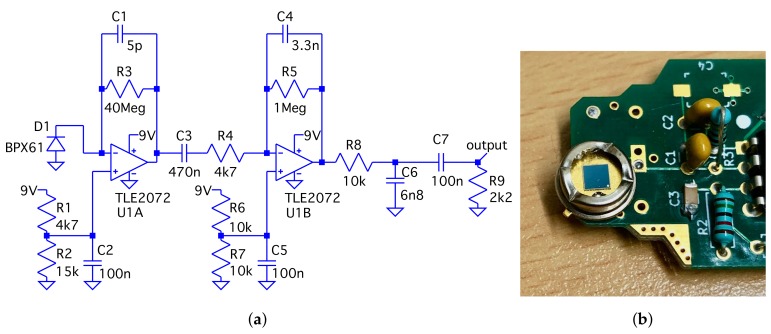
PIN diode and its amplifier circuit: (**a**) A dual-stage amplification circuit optimised for full waveform sampling at low audio sampling rates. (**b**) The surface of the silicon diode chip is exposed to radiation directly by removing the glass cover of the metal can package. The glass was broken off by carefully applying pressure using a wire cutter at four positions around the metal case.

This detector is based on the positive-intrinsic-negative (PIN) layer combination type photodiode BPX61, intended for sensing light in the visible range [14]. Photodiodes have been proposed as early as in the 60s–70s for detection of radioactivity. In 1983 Dousse and Rhême showed that this diode, manufactured at the time by Siemens, was capable of performing precise α-spectrometry with a peak resolution of 18 keV full width at half maximum (FWHM) under vacuum conditions and by using advanced nuclear physics instrumentation [15]. After the Chernobyl nuclear disaster in 1986, do-it-yourself (DIY) journals published electronic circuits for amateurs and enthusiasts to monitor the local radiation environment [16]. Our circuit follows the principle idea behind the PocketGeiger project [17], which was created after the Fukushima Daiichi nuclear disaster in order to support citizen science initiatives with a low-cost and easy to build instrument. The BPW34 family of PIN-type photodiodes, currently manufactured by Osram and Vishay [18], investigated here and in other citizen science projects [19] features a ∼10 μm thick intrinsic layer (at 0 V bias) [20,21]. This layer is sensitive to ionising radiation when operated in complete darkness (a metal case is used to shield the detector from light and electromagnetic interference radiation) and can be further increased by applying a reverse bias voltage. Combined with a high amplification factor (>10${}^{7}$) the signal pulses are compatible with the external microphone input of headset sockets found on smartphones. In this mode, the PIN diodes are typically used only as counters for ionising radiation. The presented circuit is, in contrast, optimised to measure the energy spectra of impinging particles and is shown in Figure 2a. We selected an improved low-noise operational amplifier, different time constants, and another PIN diode to adapt the small signals of electrons, as well as the large pulses of α-particles to the typical signal-recording conditions for headset microphones: ±1 V and 48 kHz sampling rate. This allows full sampling of the pulse waveforms, which are ∼1 ms wide at the output of the circuit. The sensitive area of the diodes is 7.02 mm^2^ in each case.

**Figure 3:**
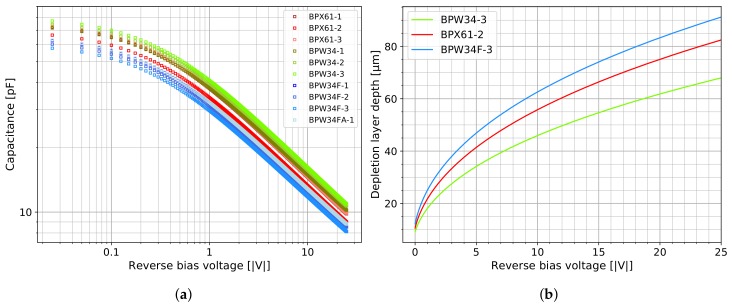
The C-V measurements of 10 diodes (four different variants) shown in (**a**) have been conducted with the Keysight B1500A at 1 MHz, 50 mV AC amplitude, parallel configuration, at 20 ${}^{\circ }C$, and using a custom-built closed metal fixture. The depletion depth dependance on the reverse bias voltage in (**b**) is based on three of the C-V curves (min, max, and average) by applying Equation (1).

According to the datasheets [14,18], the silicon sensors inside the transparent BPW34 and BPX61 diode packages have the same optical parameters for detecting the visible spectrum of light. The similarity of the silicon chips was confirmed by characterising 10 different diodes with capacitance versus voltage (C-V) measurements, as shown in Figure 3a. Across the measured reverse bias voltage range of 0 V to −25 V, the capacitance variation lies within ±15%, which can be well related to standard doping variations in the manufacturing processes. Following the procedure described in [20] (p. 3, Section III B), doping profiles generated from our C-V measurement curves (cf. the diode characterisation section in the data analysis repository [22]) show on average an effective doping concentration of $4\times 10^{12}$ cm^−3^, which is half of the values reported for the BPW34F in 2007, [20] (p. 4, Figure 5). Bayhan et al. report an even higher value for the BPW34 in 2016 [21] using a different method. These differences could be explained by manufacturing changes introduced in the last 10 years or an indication that the actual process variations are larger than the range covered by the 10 diodes we measured. The BPW34 diode family is available from two manufacturers and features different plastic packaging acting as filter for various light ranges (we investigated the BPW34 and BPW34F models from Vishay and the BPW34FA from Osram). The BPX61 diode is currently only available from Osram. It is more expensive due to its metal case compared to the plastic packaged BPW34 but features a glass window that can be easily removed to make the detector sensitive for α-particles (cf. Figure 2b). If detection of α-radiation is not of interest and rather generic radioactivity counting capabilities are desired (cf. [17,19]), the BPW34F is most suitable since its black epoxy packaging absorbs most of the visible light spectrum and simplifies necessary shielding. The width of the depleted region of a PIN diode under reverse bias conditions can be modelled with the equation for parallel plate capacitors [20] (p. 4, Equation (2)). Depending on the applied voltage, the permittivity of silicon ϵ and sensitive area of the diode *A*, the depletion layer width *d* is defined by the following formula:

(1) $$d\left(V\right)=\frac{\epsilon \epsilon_{0}A}{C(V)}.$$

Equation (1) was used to plot the depletion layer width as a function of the applied reverse bias voltage in Figure 3b. The minimum, maximum and one average C-V curve were selected to show the range of depletion layer width within the sampled lot of diodes. When using a rechargeable 9 V NiMH block battery, with a nominal voltage rating of 9.6 V and typical operational voltage range of 9 V to 11 V, the amplifier circuit in Figure 2a will provide a reverse bias voltage of about 8 V to the diode. Under this condition, the depleted layer width (or thickness) corresponds to (50±8) μm. Knowing this width (or thickness) is important as it defines the size of the region sensitive to ionising radiation. Depending on the impinging particle type and energy, physical limits can be inferred which define the detectable particle spectra. Based on the low detection probabilities stated in Figure 1, we consider the sensitivity towards X-rays and γ-photons of the presented diode detector as negligible and rather optimised the amplification and signal processing for a broad energy range up to several MeV with a correspondingly increased minimum detection threshold. Charged particles, however, interact strongly with silicon and will reach on average a certain depth depending on their energy: The projected range of electrons and penetration depth of α-particles in 50 μm of silicon corresponds to about 200 keV and 8 MeV respectively [11,12]. This allows us to predict that electrons from $\beta^{-}$-decays occurring in natural sources of radioactivity will be only partially detected by the diode sensor. α-particles from environmental radon should be almost completely absorbed since their characteristic energies range from 5.3 MeV to 8.8 MeV (^220^Rn and ^222^Rn progeny). These findings permit the use of the presented DIY particle detector as a spectrometer for naturally occurring sources of α- and $\beta^{-}$-radiation.

The developed software is available in the following repository together with the corresponding electronic hardware design files, original data of discussed measurements and supplementary information for conducting own measurements: [22,23]. The software consists of two parts: Part one records all pulse waveforms greater than the noise threshold at the audio input of a mobile device and distinguishes in real-time between α- and electron particles based on the pulse size. The second software part examines the pulses from a previous measurement run by analysing the full waveform data off-line. It applies an energy calibration to the arbitrary units of the measured pulse amplitude and plots the resulting energy spectra.

### 2.2. Hybrid Pixel Detector

The hybrid pixel detector used in some of the following measurements is based on the Timepix readout chip [24], with a sensitive area of 1.4 cm×1.4 cm divided into 256 pixels×256 pixels. The circuit embedded in each pixel measures over a wide energy range depending on settings and applied calibration, in our case from 4 keV to about 2 MeV per pixel. The calibration has been done with low energy photon sources up to 60 keV [25], the accuracy for higher energetic traces of α-particles is, therefore, impacted with an error of up to 20% (as measured for 10 MeV lithium ions in [26]). The tablet-based iPadPix prototype, summarised in [27], records the following measurements using a 300 μm thick silicon sensor attached to the Timepix chip. The recorded data is transmitted via WiFi to a computer and further analysed using python scripts. Compared with the PIN diode detector described in Section 2.1, the main differences are increased energy sensitivity towards the lower end, distinction between a larger number of different particle types, larger sensitive area and volume. The sensor surface of the hybrid pixel detector is about 28 times larger and the sensitive layer six times thicker compared to the investigated photo diodes. The sensitivity towards γ-photons is still rather limited (cf. Figure 1). Different particle types are discriminated according to the geometrical shape and energy profile of recorded ionisation patterns in the pixel matrix following the categories and algorithm established in [27]. Since these hybrid detectors are specifically designed to sense ionising radiation, they do not suffer from the same limitations as imaging sensors of consumer devices employed in those scenarios.

## 3. Results

In this section, we compare the usage of our two mobile detectors in different experiments and measurement scenarios which are all conducted at ambient air pressure conditions. This is important in the educational and citizen science context where vacuum pumps and chambers are generally not available. While α-spectrometry is typically performed in vacuum, we include an energy calibration for the diode detector documenting its sensitive range and resolving power when operated under normal ambient air conditions.

### 3.1. Energy Calibration of the DIY Particle Detector

**Figure 4:**
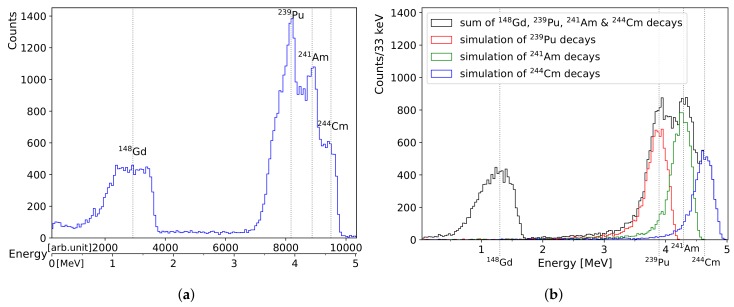
Comparing the measured response of our DIY particle detector to a reference α-source on the left with a simulation using the AASI software [28] on the right. The lower axis in (**a**) shows the energy scale after applying the calibration from Figure 5. The simulation parameters applied in (**b**) take into account the estimated ambient air density during the measurement (1.123 kg/m^3^) and corresponding geometries of the source (19 mm diameter), the diode and their positions (distance of 11 mm).

A mixed reference α-source was placed 11 mm in front of the BPX61 diode. It features each 1 kBq of ^148^Gd, ^239^Pu, ^241^Am and ^244^Cm, electroplated on top of a thin layer of palladium. After 49 min of recording time, 46,596 pulses are identified and the maximum pulse amplitudes are plotted in histogram Figure 4a. The integrated pulse areas can be evaluated alternatively. However, we found this approach is more affected by pile-up if the source activity is too high. In case of the combined 4 kBq reference α-source, some pulse pile-up is already observable.

The monoenergetic α-particles emitted from ^148^Gd are about 2 MeV lower in energy compared to the other reference isotopes and, therefore, most affected by energy straggling. This leads to the asymmetric and broad bump on the left side of the histogram. The characteristic α-energies of the other three isotopes are close together and demonstrate a practical resolution limit of about 300 keV between the nearest relevant lines of ^241^Am and ^244^Cm. Table 1 lists the relevant radioactive isotopes mentioned in this article and their properties.

**Figure 5:**
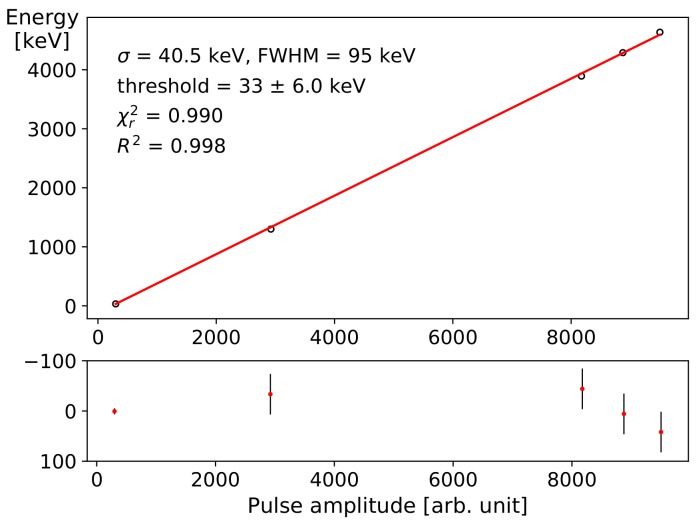
Calibration curve for the reference α-source, fitting the simulated energy peaks to the pulse amplitudes in the recorded histogram. The lower difference plot shows the deviations of the pulse amplitude data from the linear fit including error-bars. The standard deviation is derived from the FWHM value which was used to link the AASI simulation with the recorded data: σ≈FWHM/2.355.

Measurements of α-particles outside a controlled vacuum are typically more limited by the interactions with air molecules and the resulting straggling than by the accuracy and resolution of the detector. In order to match detector pulse histograms with the correct energy spectrum of the reference source, the freely available simulation software AASI is used [28]. Figure 4b shows a simulated spectrum of the mixed reference source reproducing the conditions during the measurement depicted in Figure 4a. The shapes of the peaks and absolute count rates can be only roughly reproduced in the simulation since so-called peak tailing occurs. It describes the asymmetric broadening of the α-peaks towards lower energies by a convolution of a main Gaussian with additional exponential envelopes applied on the left side of each energy peak. AASI allows to model these exponential envelopes with several parameters. The simulation was manually optimised to reassemble the measured peak shapes by tweaking the tailing parameters. After matching the α-peak centroid energies in the measured histogram with the simulation, the energy calibration shown in Figure 5 is obtained. An X-ray machine is used to measure the minimum sensitivity of the detector by increasing the acceleration voltage step-wise until pulses appear. This threshold value is determined as 33 keV with an estimated error of ±6 keV. The FWHM error of the detected α-source peaks is estimated at 95 keV each by satisfying the $\chi_{r}^{2}$ test metric applied to the linear fit. The same FWHM value was used in the AASI simulation settings for modelling the detector resolution. The actual energy resolution of the diode (cf. [15]) and noise contribution of the electronics is expected to be better but the ambient air pressure accounts for so much straggling that it is difficult to identity the true centroid positions within broadened peaks. An additional complication is introduced since the ^148^Gd α-spectrum is the only monoenergetic one. The other isotopes of the reference source produce several characteristic α-decay energies, which also broadens each measured peak, and the resulting superposition cannot be resolved without applying advanced compensation techniques for the peak tailing. Table 1 shows the individual decay energies of ^239^Pu, ^241^Am, and ^244^Cm are too close together in comparison with the estimated FWHM detector resolution of 95 keV (general rule: min. peak distance ≥3×FWHM [29]). The relatively large FWHM value, therefore, primarily accounts for uncertainties of the physical boundary conditions: distances and cross sections of passive and active layers and their corresponding density values.

### 3.2. Experiments on Airborne Radon Isotopes

**Figure 6:**
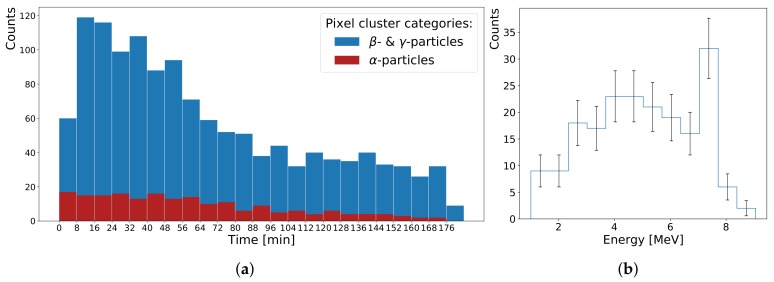
Measuring radon progeny with a balloon: (**a**) While the α-decay rate decreases monotonic, the $\beta^{-}/\gamma$ particle rate builds up as products of the short lived polonium parent. The last bin on the right shows the background rate, without balloon. (**b**) The sparse energy spectrum of the α-particles corresponds to the different isotopes of polonium. Its upper limit suggests energies are overestimated given the air gap between balloon and pixel detector surfaces. The correction from [26] was applied.

Radon is a radioactive gas which diffuses from natural traces of thorium and uranium in the soil into the air. Using an electrostatic charged party balloon a sample of radon progeny, ^218^Po and ^216^Po, is collected for 15 min in a closed room like an office or classroom [1,30]. Figure 6a shows a measurement using the hybrid pixel detector. By applying the algorithm from [27] different particle categories are identified from the recorded pixel cluster patterns. After collection of radioactive isotopes the balloon is carefully deflated and placed 1 cm in front of the detector.

This experiment is less suitable for the diode detector due to its small sensitive area, which cannot be compensated with longer measurement periods. The main duration of activity is about 3 h; after that the averaging intervals must be increased considerably to compensate for background radiation (cosmic radiation, nearby decaying radon, and other naturally occurring radioactive isotopes, like ^40^K). The staged shape of the falling envelope of the combined $\beta^{-}/\gamma$-decay rate can be attributed to the half-lives of the first (^214^Pb) and second (^214^Bi) progeny of ^218^Po (cf. Table 1). The corresponding α-spectrum in Figure 6b is given only for comparison with the other spectra taken with the diode detector. In this case, the statistics are too low for a detailed peak analysis. Moreover, the observed energy straggling of α-particles is even greater compared with the diode measurements. Due to the much larger sensitive area (×28), possible paths lengths of the α-particles bear a larger variance.

### 3.3. Experiments on Edible Potassium Salt

**Figure 7:**
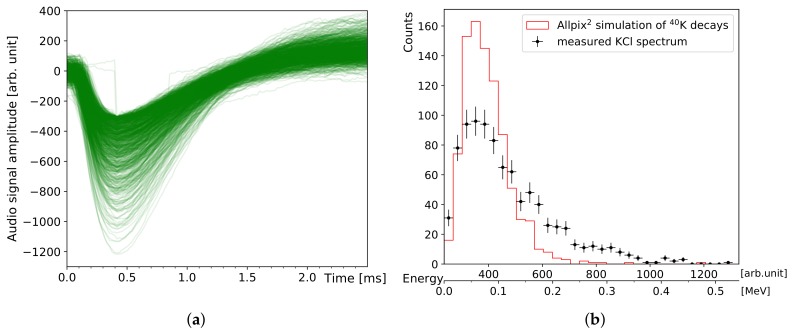
Measurement and simulation of the DIY particle detector response to KCl: (**a**) The negative component of the circuit ouput pulses is ≈1 ms wide, the amplitudes are proportional to the energy deposited by ionising radiation. Two pulses are truncated (sharp vertical edges) due to incomplete sampling. (**b**) Histogram of the pulse amplitudes recorded in Figure (**a**), including energy calibration on the lower x-axis. The red line shows a simulation for the same number of decays from pure ^40^K.

Naturally occurring potassium is always accompanied by the radioactive isotope ^40^K, which renders animals, humans, plants, and many kinds of food, such as nuts and bananas, slightly radioactive. Normal table salt, NaCl, can be substituted with freely available KCl. Its activity provides a harmless $\beta^{-}$- and γ-source for estimating the efficiency of radiation detectors (1 g(KCl)≙16.25 Bq, [31]). Figure 7a,b show measurements and simulations of 9.44 g KCl, corresponding to a total activity of 153.4 Bq, with the diode detector. It is a flat piece made of crystallised KCl powder with a maximum thickness of 1 cm and an area of ≈3 × $3{\mathrm{cm}}^{2}$. Within 707 min, 906 pulses are recorded corresponding to a count rate of 1.28 cpm: distinctively higher than the average background rate of 0.06 cpm with this detector (in a typical 0.1 μSv/h environment of low-background radiation). The simulation in Figure 7b is calculated using Allpix${}^{2}$, an open source simulation framework which permits simulation of a broad range of semiconducting detector setups and radiation sources [32]. It simplifies usage of the underlying Geant4 toolkit, which is a powerful but complex software for simulating a large variety of particle interactions in matter. The employed simulation parameters are based on the results from the energy calibration and show a typical peak shape which is impacted by the detector threshold towards low energies on the left. The peak maxima of the simulation and measurement occur in the same region but the shape of the latter is broader in comparison, which can be explained by three factors: (1) Internal scattering within the KCl block is not taken into account, which decreases the maximum β-decay energy of 1.3 MeV emitted from ^40^K. This shifts the energy range of source electrons closer to the range which can be fully absorbed in the diode because energetic electrons with E ≥ 1 MeV behave like minimum ionising particles in silicon, depositing small amounts of energy well below the detector threshold. (2) For small charge pulses close to the noise level, the amplification circuit can be less linear than assumed in the calibration. Further reference measurements with monoenergetic electron sources would be necessary in this case. (3) The depleted region of the silicon diode is less homogeneously doped than assumed, and its depth could, therefore, deviate from the estimated range of values.

A practical threshold can be inferred from the measured and simulated KCl spectra in order to distinguish between α-particles and electrons at about 500 keV. This corresponds to a pulse amplitude of ≈1243 and an integrated pulse area of ≈35,425. The mobile software uses this value in order to discriminate the pulses in real-time into one of both particle categories and to deduce corresponding count rates of α-particles and electrons per time interval.

**Figure 8:**
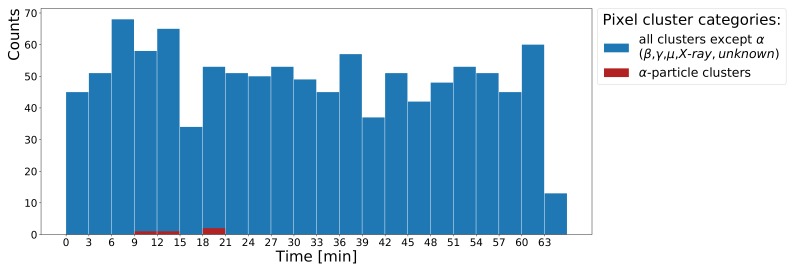
Histogram of KCl radioactivity measured for 1 h using the hybrid pixel detector. The last bin on the right shows the background rate without KCl. Four α-particles are detected in the first half of the measurement as part of indoor radioactivity caused by radon.

Since the diode area is more than hundred times smaller than the KCl block, it is more adequate to evaluate the efficiency with the hybrid pixel detector. Figure 8 shows the corresponding measurement of 1079 detected particles in the $\beta^{-}/\gamma$ category within one hour. If we ignore γ-radiation due to the low sensitivity of the thin sensor layer in the hybrid detector assembly, only 89.28% of ^40^K decays are relevant because they undergo $\beta^{-}$-decays. Taking this into account, the detection efficiency is estimated as 1079/(60×60 s×153.4 Bq×89.28%)=0.22%. This low value is on the one hand due to internal absorption of the majority of the radiation before it leaves the KCl block and on the other hand because only a fraction of the isotropic decay distribution is covered by the sensitive area of the hybrid pixel detector.

### 3.4. Experiments on Radioactivity from Natural Uranium and Its Isotopes

**Figure 9:**
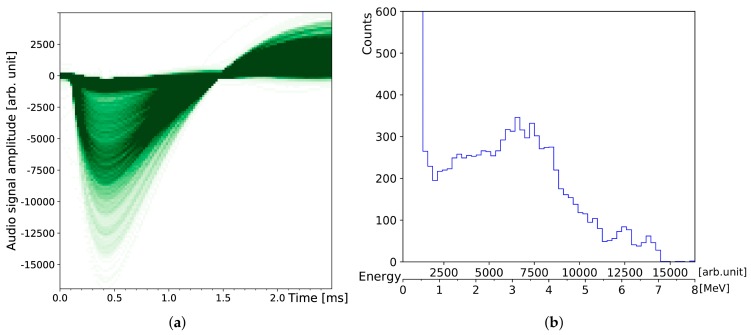
Measurement of a radioactive columbite stone using the DIY particle detector: Compared to Figure 7a, the 56,147 detected pulses cover a much larger spectrum of values. The pulses on the left Figure (**a**) are superimposed using transparent ink in order to reveal an alternative representation of the maximum pulse amplitude distribution depicted in the energy histogram on the right (**b**).

In this section we measure a small piece of columbite/coltan mineral sold by a German company offering resources for physics education. The activity is rated at 100 Bq and, therefore, well below the applicable exception limits of most governments. The stone features trace amounts of natural occurring uranium ore (^238^U) on its surface, which are measured by the diode detector. Its darkest spot was measured as close as possible, touching the metal case of the diode at a distance of about 2 mm in front of the silicon sensor, as shown in Figure 9a,b.

The energy spectrum shows two main characteristic areas which can be distinguished. The steep edge on the left corresponds to $\beta^{-}$-decays reaching a maximum detected energy of about 500 keV. This confirms the result of the previous KCl measurement. The larger area on the right corresponds to α-decays. This α-spectrum reveals several distinctive areas after 70 h of combined measurements (across five nights, each measurement was started with a fully charged battery) since the activity is very low and most of the radiation is partially absorbed inside the stone before it reaches the outside. The α-emitters which are most likely to be detected from ^238^U are radium (^226^Ra together with its direct progeny ^222^Rn, mainly accounting for the broad peak across 3 MeV to 4 MeV in Figure 9b) and all of its rather short lived progeny with characteristic peak energies from 4.8 MeV to 7.7 MeV. The two small peaks on the right can be attributed to the high-energy α-particles of the polonium isotopes. Similar results are expected from measuring minerals containing natural thorium (^232^Th), such as black monazite sand.

**Figure 10:**
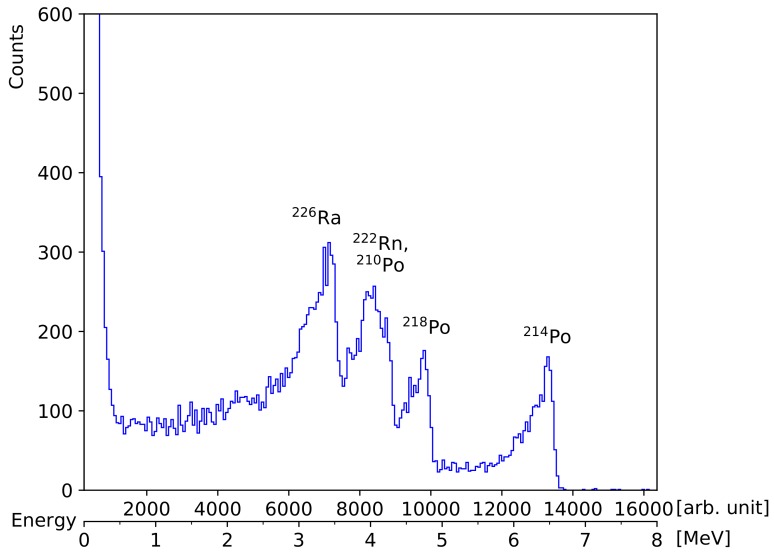
Energy histogram of a radium painted watch hand recorded with the DIY particle detector. Four peaks are clearly identified, even without guidance by a simulation since most characteristic α-energies are further apart in this measurement than the straggling broadens them.

A comparatively well defined α-spectrum from ^226^Ra is shown in Figure 10. The specimen for this measurement is a small clock-hand painted with radium paint, as it was a customary method for creating self-illuminating watches predominantly in the first half of the 20th century. It was placed 11 mm in front of the diode detector under the same conditions as the measurement of the reference α-source show in Figure 4a. Four peaks are well distinguished since the source is only a thin layer of paint featuring low internal absorption in comparison to the columbite stone. The energies correspond to ^226^Ra, ^210^Po, ^222^Rn, ^218^Po, and ^214^Po in increasing order. The third peak from the right represents an overlap of ^222^Rn and ^218^Po since their characteristic energies are so close together (cf. Table 1). All four centroid energies are about 1.3 MeV lower than their characteristic value, which corresponds well with the expected energy loss within the ambient air in front of the detector. Similar well defined spectra can be expected from small debris particles or dust in the case of a nuclear fallout (e.g., Chernobyl). Explosive releases of nuclear fuel from a power plant, such as ^239^Pu (which was used for calibrating the detector in Section 3.1) or ^235^U, could be identified by their characteristic α-peak energies. In the case of such a nuclear disaster, our DIY particle detector would permit gaining a deeper insight into the event. Greatest care should be taken with such measurements to avoid any possibility of contamination when handling brittle radioactive material.

## 4. Discussion

This contribution presents several examples of usage of a mobile device-based silicon pixel detector, iPadPix, and its single pixel counterpart based on a photodiode for the detection and investigation of ionising radiation in the environment. As Table 1 shows, possible applications cover α-, $\beta^{-}$-, γ-radiation from radioactive sources to be encountered in all states of matter. Applications to water samples, not presented here, include the possibility of detection (and monitoring) of radioactivity in tap, source, or bottled water after filtration. As amounts of natural uranium and thorium in water are in the order of few μg/L, the larger sampling surface and increased sensitive volume of iPadPix is favourable in this case.

In comparison with previous work using smartphone cameras re-purposed as radioactivity detectors, the present approach has several advantages. First, better reliability: the former exhibit in general large systematic errors ([5] reports up to 50%) due to geometric variances between different camera sensors and software calibration issues (cf. Section 1).

Second, increased sensitivity: The sensitive layer thickness in smartphone cameras is typically in the order of a tenth of the hybrid pixel detector and within a fifth of the diodes which we used: [7] report an estimation of 15 μm to 27 μm for a smartphone from 2011, modern digital cameras are constructed with sensor depths close to 10 μm, which is sufficient to absorb visible light (cf. absorption probabilities shown in Figure 1). The silicon detectors we propose feature considerably larger and known sensitive volumes: BPX61 & BPW34 depletion layer thickness confirmed at (50±8) μm, which allows setting physical boundaries on possible particle measurements and energy ranges. Our measurements demonstrate sensitivity to sources of natural radioactivity down to the order of 100 Bq, one order of magnitude below e.g., activities of commercial radioactive sources typically used in school settings ([5] used 45 kBq ^90^Sr and 370 kBq ^137^Cs isotope generator). This increased sensitivity enables also shorter measurement intervals in comparison with smartphone imaging sensors (cf. [6]).

Third, particle discrimination and measurement of energy spectra: Different particle types, such as α-particles and electrons from $\beta^{-}$-decays, can be discerned directly by their detectable signal. For the presented DIY diode detector, a threshold of 500 keV was characterised with the help of simulations to serves as a discriminator between α- (energy above threshold) and $\beta^{-}$-decays (energy below threshold). The sensor surfaces are almost directly exposed, with only a thin passive layer required to connect the silicon bulk material electrically. This reduces the lower energy limit considerably compared with smartphone cameras where several millimetres of plastic and glass must be traversed by ionising radiation, which leads to increased absorption and scattering before the sensitive volume is reached. Moreover, smartphone cameras, as well as Geiger-Müller tubes, cannot measure energy spectra of ionising radiation directly (only indirectly by shielding and application of specific absorption materials). The energy range of our diode detector was verified from a minimum detection threshold of 33 keV up to 8 MeV, with a practical resolution of 95 keV FWHM when measuring alpha particle energies and while being operated under ambient air pressure levels. We demonstrated that an additional vacuum machine—which is typically considered necessary for α-spectrometry in professional contexts—is not needed to clearly identify naturally occurring radioactive isotopes, such as radium and its progeny, with low-cost tools. This is an important aspect for mobile outdoor measurements in the field.

Fourth, the availability and openness of the software and hardware presented here: With closed-source software, such as the *RadioactivityCounter* mobile application mentioned in Section 1, a lack of updates for compatibility with recent devices limits its usefulness. This poses a special concern in education and citizen science contexts where utilisation of modern personal devices is essential. In contrast, the hardware design files and software sources of the presented DIY particle detector are made available respectively under the permissive CERN open hardware license (CERN OHL) and the BSD license [22]. The data acquisition part, running on the smartphone is written using modern HTML and Javascript programming interfaces that have historically provided good compatibility with future mobile web browsers. The analysis scripts are written in python, a programming language becoming increasingly popular in data science and education environments. Software and hardware parts required to build iPadPix are detailed in [25,27]. Its Timepix-based detector component is about one order of magnitude more expensive compared with the low-cost photodiodes, which limits is accessibility. Although, other energy-resolving devices like professional α- and γ-spectrometers are equally expensive and typically restricted to detecting only one particular radiation type per device.

From an educational perspective, the uses of silicon sensor technology presented here allow for (i) classical experiments with less expenses and fewer limitations (e.g., regarding energy spectra and particle discrimination) at the same time, and (ii) for several novel experiments. Classical experiments for the classroom require either bulky or expensive apparatus like Geiger-Müller counters [1,33,34] or photo-multipliers [35,36]. The mobile and low-cost character of the presented detectors, combined with their wide range of applications, provides novel teaching and learning opportunities for a series of important educational objectives in the context of radiation and radioactivity:

1) Insight into the ubiquitous nature of radioactivity occurring also in our natural environment (see Table 1). Regarding health and safety issues is not only the answer to the question of dose (how much radiation?) decisive, but rather a deeper understanding about different particle types and their nuclear physics properties (what kind of radiation?).
2) Experiential learning concerning common confusions and misconceptions about radiation and radioactivity, such as (i) the “non-naturalness” idea that all radiation and radioactivity is artificial ([37], see also see point 1) above); (ii) the “contamination” idea that irradiation of food or a person, e.g., by X-rays, results in radioactive contamination [38,39,40].
3) Taking into account evidence from science education research about science contexts interesting for young people of high school age. Physics is meeting notoriously low interest among high-school students, in particular among girls [41]. However, the very same physics content can be perceived with very different interest, depending on the kind of context (i.e., area of application) it is linked with. Biomedical applications belong to the context establishing the highest interest among girls, much higher than technological applications: e.g., for the content of hydrostatic pressure the heart as “blood pump” versus a pump to extract petrol from great depths; for boys interest in both contexts is similar and rather high [42,43]. Empirical evidence for the effectiveness of interventions of taking account of these findings has been provided, e.g., by [44,45]. Using smartphone and tablet-based sensing of environmental radioactivity offers such a biomedical context and measuring properties of α-particles in particular provides links to the physics of modern hadron therapy used in treatment of cancer. Moreover being of high environmental and societal importance, another factor supporting physics interest among girls [43]. Another topic shown to be of very high interest for secondary level students is astronomy and astrophysics [46]. Within the last five years, it was also explored with the help of smartphones, from a basic [47] to research level [7,8]. This further underlines potential and perspectives of using mobile sensors beyond biomedical applications.

Besides education, recent developments, in particular after the Fukushima Daiichi nuclear disaster, have led to citizen science projects using sensors of portable devices for radiation monitoring [2,17,48]. Important societal consequences of these developments were discussed very recently in a related journal [49]. The present contribution shows how mobile device-based sensors can provide means for continuous monitoring and energy-sensitive investigation of ambient radioactivity. We believe that the increased functionality—and in case of the diode sensor, low-cost nature, and availability—allow for widespread use by individuals and citizen science groups. Improved physical insight about environmental and health protection issues related to radioactivity is expected as we shift the toolkit from mere counting towards discerning different kinds and energy ranges of ionising radiation. As Beser quotes Franken in 2016 [50] regarding the Fukushima incident, “[...], we were worried, but we couldn’t get our hands on anything.[...] People were looking for information, so we saw that there was a need. Our plan became: get information, put it together and disseminate it.” Mobile radiation sensors with spectrometric capabilities, as presented, appear as a promising way to contribute to this important task in the future.

Our next steps will be to further investigate other sources of natural radioactivity, such as cosmic particle showers, using similar approaches.

## Figures and Tables

**Table 1:** Properties of measured isotopes, mostly from naturally occurring radioactive materials (NORM). From the ^238^U decay chain only the relevant short-lived isotopes are included. For $\beta^{-}$-decays, the maximum energy of each continuous spectrum is stated. The ^232^Th decay chain is omitted since the half-life of its radon progeny ^220^Rn is orders of magnitude lower compared to ^222^Rn and, therefore, less relevant in our measurements. The progeny of the ^232^Th series has in general similar characteristic decay energies in comparison to the ^238^U series.

Isotope	Half-Life	Main Decay	Energies (≥1% Probability)	Progeny	Occurrence in Nature
^40^K	1.3 Gy	$\beta^{-}$ (89.28%)	1.311 MeV	^40^Ca	living beings, food, stones
^148^Gd	74.6 y	α	3.183 MeV	^152^Dy	- (synthetic)
^214^Bi	19.9 m	$\beta^{-}$	787 MeV to 1540 MeV	^214^Po	aerosols, water, stones
^214^Pb	26.8 m	$\beta^{-}$	672 MeV, 729 MeV	^214^Bi	aerosols, water, stones
^210^Po	138.4 d	α	5.407 MeV	^206^Pb	aerosols, water, stones
^214^Po	163.7 μs	α	7.687 MeV	^210^Pb	aerosols, water, stones
^218^Po	3.098 m	α	6.002 MeV	^214^Pb	aerosols, water, stones
^222^Rn	3.823 d	α	5.490 MeV	^218^Po	air, water, stones
^226^Ra	1600 y	α	4.784 MeV, 4.601 MeV	^222^Rn	aerosols, water, stones
^239^Pu	1.3 ky	α	5.157 MeV, 5.144 MeV, 5.106 MeV	^235*m*^U	nuclear fallout (synthetic)
^241^Am	432.8 y	α	5.486 MeV, 5.443 MeV	^237^Np	- (synthetic)
^244^Cm	18.1 y	α	5.805 MeV, 5.763 MeV	^240^Pu	- (synthetic)
